# Altered oxidative stress and antioxidant defence in skeletal muscle during the first year following spinal cord injury

**DOI:** 10.14814/phy2.14218

**Published:** 2019-08-27

**Authors:** Mladen Savikj, Emil Kostovski, Leonidas S. Lundell, Per O. Iversen, Julie Massart, Ulrika Widegren

**Affiliations:** ^1^ Faculty of Medicine University of Oslo Oslo Norway; ^2^ Department of Research Sunnaas Rehabilitation Hospital Nesodden Norway; ^3^ Department of Physiology and Pharmacology, Section for Integrative Physiology Karolinska Institutet Stockholm Sweden; ^4^ Department of Nutrition, Institute of Basic Medical Sciences University of Oslo Oslo Norway; ^5^ Department of Haematology Oslo University Hospital Oslo Norway; ^6^ Department of Molecular Medicine and Surgery, Section for Integrative Physiology Karolinska Institutet Stockholm Sweden

**Keywords:** Atrophy, oxidative stress, skeletal muscle, spinal cord injury

## Abstract

Oxidative stress promotes protein degradation and apoptosis in skeletal muscle undergoing atrophy. We aimed to determine whether spinal cord injury leads to changes in oxidative stress, antioxidant capacity, and apoptotic signaling in human skeletal muscle during the first year after spinal cord injury. *Vastus lateralis* biopsies were obtained from seven individuals 1, 3, and 12 months after spinal cord injury and from seven able‐bodied controls. Protein content of enzymes involved in reactive oxygen species production and detoxification, and apoptotic signaling were analyzed by western blot. Protein carbonylation and 4‐hydroxynonenal protein adducts were measured as markers of oxidative damage. Glutathione content was determined fluorometrically. Protein content of NADPH oxidase 2, xanthine oxidase, and pro‐caspase‐3 was increased at 1 and 3 months after spinal cord injury compared to able‐bodied controls. Furthermore, total and reduced glutathione content was increased at 1 and 3 months after spinal cord injury. Conversely, mitochondrial complexes and superoxide dismutase 2 protein content were decreased 12 months after spinal cord injury compared to able‐bodied controls. In conclusion, we provide indirect evidence of increased reactive oxygen species production and increased apoptotic signaling at 1 and 3 months after spinal cord injury. Concomitant increases in glutathione antioxidant defences may reflect adaptations poised to maintain redox homeostasis in skeletal muscle following spinal cord injury.

## Introduction

Onset of traumatic spinal cord injury leads to extensive skeletal muscle atrophy due to neural system decentralization and disuse (Biering‐Sorensen et al., 2009). The loss of muscle mass is rapid during the initial 2 months after injury, with a ~ 60% reduction in average fiber cross‐sectional area compared to able‐bodied controls, and an additional ~ 20% decrease in the following 4 months ( Castro et al., 1999). Muscle wasting after spinal cord injury is accompanied by decreased peripheral glucose disposal and reduced skeletal muscle mitochondrial content and fatty acid uptake (Aksnes et al., 1996; Kjaer et al., 2001). Decreases in mitochondrial capacity are associated with changes in body composition including increased adiposity (O’Brien et al., 2017), which contributes to increased risk of cardiovascular disease and type 2 diabetes (Cragg et al., 2013). Interventions targeted toward mechanisms to mitigate skeletal muscle atrophy are warranted to prevent such chronic disorders and improve the well‐being of individuals with spinal cord injury.

Skeletal muscle atrophy has been linked to increased oxidative stress, defined as an imbalance between the production and detoxification of reactive oxygen species (ROS) (Kondo et al., 1991). Multiple sources are responsible for excessive ROS production in atrophying skeletal muscle. Predominantly, ROS production occurs within the dysfunctional mitochondria through electron transport chain complexes I and III (Min et al., 2011; Gram et al., 2015). Contribution to ROS production is also made by nicotinamide adenine dinucleotide phosphate (NADPH) oxidase (NOX) isoforms. In rodents, protein content of NOX4, localized at the sarcoplasmic reticulum, increases following spinal cord injury, while NOX2 activity at the sarcolemma contributes to angiotensin II‐induced muscle atrophy (Liu et al., 2017; Kadoguchi et al., 2018). Finally, ROS production by xanthine oxidase (XO), during the final steps of purine degradation, increases during skeletal muscle atrophy in rodents (Kondo et al., 1993; Derbre et al., 2012). Thus, loss of muscle mass is accompanied by increases in ROS production both within and outside the mitochondria.

Conversely, several lines of antioxidant defences are poised to counteract excessive ROS production. [Cu‐Zn]‐superoxide dismutase (SOD1) in the cytoplasm and [Mn]‐superoxide dismutase (SOD2) within the mitochondria convert superoxide radicals into hydrogen peroxide (Fukai and Ushio‐Fukai, 2011). Superoxide also reacts with nitric oxide, in a concentration‐dependent reaction, producing peroxynitrite (Huie and Padmaja, 1993). Peroxynitrite can be further decomposed to nitrite by mitochondrial complex IV, while hydrogen peroxide is further detoxified into molecular oxygen and water by catalase and glutathione peroxidase (GPx) (Kondo et al., 1993; Pearce et al., 1999). GPx utilizes glutathione (GSH) as a substrate, converting it to glutathione disulfide (GSSG), which is restored to its reduced form by glutathione reductase (GRx). Activity of these antioxidant enzymes increases in response to excessive ROS production during immobilization‐induced atrophy in rat skeletal muscle (Kondo et al., 1993). Hence, loss of muscle mass is accompanied by a compensatory adaptation in antioxidant capacity in parallel to increases in ROS production.

While ROS play a role in the beneficial adaptive response to exercise in healthy skeletal muscle (Gomez‐Cabrera et al., 2008), ROS also have a causative role in the development of skeletal muscle atrophy (Kondo et al., 1993; Min et al., 2011; Derbre et al., 2012). Oxidative stress damages cellular structures. Nonenzymatic lipid peroxidation by ROS leads to production of lipid aldehydes, such as 4‐hydroxynonenal (4HNE), which can interfere with protein function and lead to activation of apoptotic pathways (Dalleau et al., 2013). Oxidative stress promotes damage to protein, such as carbonylation, rendering proteins more susceptible to degradation (Smuder et al., 2010). Furthermore, exposure to exogenous ROS increases the expression of genes involved in protein catabolism, while simultaneously interfering with protein synthesis (O’Loghlen et al., 2006; Dodd et al., 2010; McClung et al., 2010). Perturbation of protein metabolism lies at the core of skeletal muscle atrophy (Jackman and Kandarian, 2004). Additionally, ROS exposure triggers apoptotic pathways and may contribute to the decreased mitochondrial content during muscle atrophy (Siu and Alway, 2005). Furthermore, antioxidant treatment alleviates muscle wasting in rodent models (Kondo et al., 1991; Min et al., 2011; Derbre et al., 2012), indicating that oxidative stress contributes to atrophy. However, whether spinal cord injury leads to changes in ROS homeostasis and oxidative stress in human skeletal muscle remains unknown.

During the initial 3 months after spinal cord injury, plasma antioxidant levels are decreased, while both plasma and urine markers of oxidative stress are increased (Bastani et al., 2012). Our aim was to determine whether spinal cord injury induces changes in enzymes responsible for ROS homeostasis and consequently induces oxidative stress in the atrophying skeletal muscle. To address this question, we measured oxidative stress marker levels, as well as protein abundance of enzymes involved in ROS production and detoxification in skeletal muscle from spinal cord‐injured individuals at 1, 3, and 12 months after injury, compared with able‐bodied healthy controls.

## Materials and Methods

### Study participants

Seven individuals, four men and three women, with a complete spinal cord injury according to the American Spinal Injury Association Impairment Scale (AIS) for neurological classification of spinal cord injury (Kirshblum et al., 2011), injury range C4‐Th12, and seven male able‐bodied controls matched for age and body mass index participated in the study. Clinical characteristics of the participants are presented in Table 1. None of the participants had any known systemic diseases. Able‐bodied participants were not receiving any medications, while participants with spinal cord injury received low molecular weight heparin therapy during the first 3 months after injury, as well as spasmolytic therapy (baclofen or equivalent) when indicated. Participants with spinal cord injury received standard rehabilitative care at Sunnaas Rehabilitation Hospital, Nesoddtangen, Norway. All participants gave their written informed consent and the study was conducted according to the Declaration of Helsinki. The study was approved by the Regional Committee for Medical and Health Research Ethics at Helse Sør‐Øst Trust, Norway.

**Table 1:** Clinical characteristics.

		Able‐bodied (*n* = 7)	Spinal cord‐injured (*n* = 7)		
Age, yr (mean ± SD)		49 ± 6	43 ± 15		
			**Months after injury**		
			1	3	12
BMI, kg/m^2^ (mean ± SD)		26 ± 2	25 ± 4	25 ± 4	27 ± 3
**Injury level**			**AIS motor score (0–100)**		
Cervical (*n* = 2)	C4	n/a	8	8	16
	C6	n/a	27	27	28
Thoracic (*n* = 5)	Th3‐12	n/a	50	50	50

AIS, American Spinal Injury Association Impairment Scale; BMI, Body Mass Index; n/a, not applicable.

### Preparations of skeletal muscle biopsies

Skeletal muscle biopsies were obtained using a Bergström needle from the *vastus lateralis* of the *quadriceps femoris* muscle under local anesthesia (Lidocaine 5 mg/ml), cleaned from visible fat and blood, and rapidly frozen in liquid nitrogen. Biopsies were lysed in ice‐cold lysis buffer (137 mmol/L NaCl, 2.7 mmol/L KCl, 1 mmol/L MgCl, 20 mmol/L Tris pH 7.8, 10 mmol/L NaF, 1 mmol/L EDTA, 0.5 mmol/L NaVO_3_, 1 mmol/L PMSF, 10% glycerol (w/v), 1% Triton X‐100 (w/v), and protease inhibitor cocktail Set 1 (Calbiochem, EMD Biosciences, San Diego, CA, US)). Extracellular debris was removed by centrifugation at 12,000*g* for 10 min at 4°C, and the supernatant containing soluble material was collected. Protein concentration was determined using a commercially available Pierce bicinchoninic acid (BCA) protein assay (#23225, Thermo Scientific, Waltham, MA, US) according to the manufacturer’s instructions.

### Protein carbonylation assay

Analysis of protein carbonylation was performed as described (Trewin et al., 2015), using a commercially available kit (OxyBlot, #S7150, Merck Millipore, Burlington, MA, US). Equal amounts of protein were loaded, denatured by addition of 6% (v/v) sodium dodecyl sulfate (SDS), and carbonyl groups on protein side chains were derivatized to 2,4‐dinitrophenylhydrazone (DNP) by reaction with 2,4‐dinitrophenylhydrazine (DNPH). After 15 min at room temperature, the derivatization reaction was stopped by adding neutralizing solution and 5% (v/v) of 2‐mercaptoethanol.

### SDS‐PAGE and western blot

Western blotting was performed as described (Lundell et al., 2018). Equal amounts of protein were diluted in Laemmli buffer and separated on SDS‐PAGE (#3450124, Criterion XT Precast gels, BioRad, Hercules, CA, US). Protein was transferred to a polyvinyl fluoride (PVDF) membrane (IPVH00010, Immobilon‐P, Merck Millipore), Ponceau S staining (#P7170, Sigma‐Aldrich, St. Louis, MO, US) was performed and results were normalized to total protein per lane. Membranes were blocked using 7.5% (w/v) nonfat dry milk in Tris‐buffered saline (TBS) with Tween 20. Primary antibodies were diluted 1:1000 (v/v) in TBS containing 0.1% (w/v) bovine serum albumin and NaN_3_. Membranes were incubated in primary antibody dilutions overnight (~16 h) at 4°C under gentle agitation. The list of primary antibodies used is presented in Table 2. Species‐appropriate horseradish peroxidase‐conjugated secondary antibodies, diluted 1:25,000 (v/v) in 5% nonfat dried milk in TBS‐Tween were used, and protein amounts were visualized using chemiluminescence (#RPN2106 ECL and #RPN2235 ECL select, GE Healthcare, Chicago, IL, US). Optical density of detected bands was quantified using Image Lab v.5.2.1 (BioRad). The optical density of detected bands for protein carbonylation (molecular weights 250, 225, 100, 80, 50, 40, and 35 kDa) and 4HNE‐adducts (molecular weights 150, 100, 60 and 35kDa) were determined and the sum of the bands is presented as total.

**Table 2:** Primary antibodies list.

Antigen	Abbreviation	Size (kDa)	Manufacturer (#)^a^
4‐Hydroxynonenal	4HNE	n/a	Abcam (ab46545)
OXPHOS Cocktail	n/a	n/a	Abcam (ab110411)
NDUFB8	Complex I	18	Abcam (ab110242)
SDHB	Complex II	29	Abcam (ab14714)
UQCRC2	Complex III	48	Abcam (ab14745)
MTCO2	Complex IV	22	Abcam (ab110258)
ATP5A	Complex V	54	Abcam (ab14748)
p47phox (Ser^328^)	p‐p47 (Ser^328^)	47	Abcam (ab111855)
NADPH oxidase 2	NOX2	65	Abcam (ab80508)
NADPH oxidase 4	NOX4	67	Abcam (ab133303)
Xanthine Oxidase	XO	85	Abcam (ab109235)
Superoxide Dismutase 1	SOD1	17	Abcam (ab16831)
Superoxide Dismutase 2	SOD2	25	Abcam (ab13534)
Catalase	Catalase	64	Santa Cruz (sc‐50508)
Glutathione Peroxidase 1	GPx	22	Abcam (ab22604)
Glutathione Reductase	GRx	58	Abcam (ab16801)
SAPK/JNK (Thr^183^/Tyr^185^)	p‐JNK (Thr^183^/Tyr^185^)	46/54	Cell Signalling (9251)
SAPK/JNK	JNK	46/54	Cell Signalling (9252)
Calpain‐1	Calpain‐1	68/82	Abcam (ab28258)
Cleaved Caspase‐9	Cleaved Caspase‐9	37	Abcam (ab2324)
Caspase‐3	Caspase‐3	19/35	Cell Signalling (9662)

a Abcam, Cambridge, United Kingdom; Santa Cruz, Dallas, TX, United States; Cell Signalling, Danvers, MA, United States.

### Reduced and total glutathione detection

Glutathione content was determined using a commercially available assay kit (ab138881, Abcam, Cambridge, UK). Aliquots, normalized for protein concentration, were loaded for the reaction. To avoid assay enzymatic interference, deproteinization was performed by 1:5 (v/v) addition of 100% (w/v) trichloroacetic acid (TCA) and precipitated protein and TCA were removed by centrifugation (12,000*g*, 5 min at 4°C). The remaining TCA in the supernatant was neutralized by addition of 1M NaHCO_3_, the samples centrifuged (12,000*g*, 15 min at 4°C) and the supernatant collected for further analysis. The amount of reduced GSH and total glutathione were determined fluorometrically, and the amount of oxidized GSSG was calculated according to manufacturer’s instructions.

### Statistical analyses

The arbitrary units of optical density were normalized to the mean of the able‐bodied control group. Data are presented as mean and standard deviation (SD) with the individual data points overlaid. Statistical comparisons were made using nonparametric Mann–Whitney (Table 1) or Kruskal–Wallis tests, followed by Dunn’s multiple comparison where appropriate. *P*‐values below 0.05 were considered as significant, while 0.1 ≥ *P* ≥ 0.05 indicated trends. Statistical analyses were performed using Prism v.7.01 (GraphPad, San Diego, CA, US).

## Results

### Oxidative stress markers in skeletal muscle following spinal cord injury

**Figure 1:**
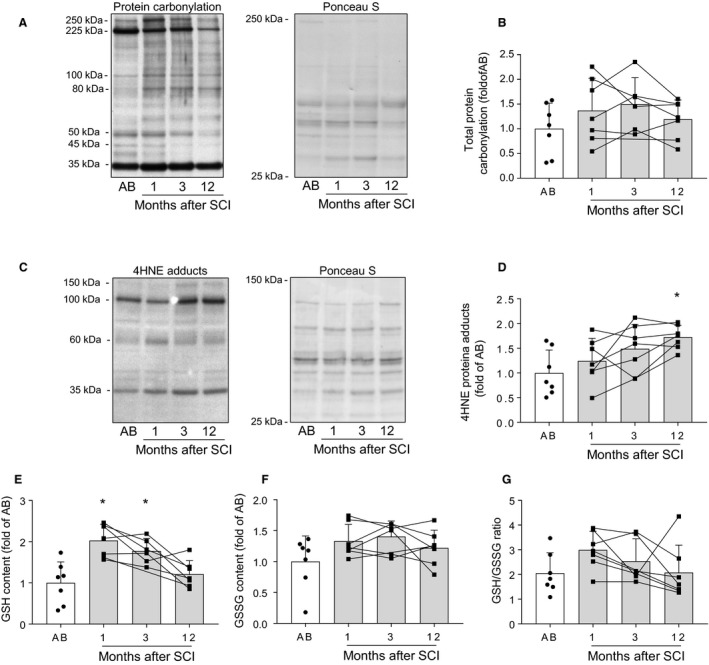
Oxidative stress markers in skeletal muscle following spinal cord injury. Representative western blot image and Ponceau S stain (A) and quantification (B) for total protein carbonylation. Representative western blot image and Ponceau S stain (C) and quantification (D) for total 4HNE protein adducts. GSH (E), GSSG (F), and GSH/GSSG ratio (G) in skeletal muscle from able‐bodied (AB, white bars) and spinal cord‐injured individuals (SCI, grey bars) at 1, 3, and 12 months post‐injury. Units are scaled to the mean of the able‐bodied controls. Data are presented as mean and standard deviation, and individual data points are overlaid. * ‐ *P* < 0.05 vs able‐bodied controls, Kruskal–Wallis followed by Dunn’s multiple comparisons test.

To assess whether spinal cord injury induces oxidative stress in skeletal muscle, we analyzed GSH and GSSG content, as well as protein carbonylation and 4HNE protein adducts as markers of oxidative damage. Total amount of carbonylated proteins was unaltered in skeletal muscle during the first year following spinal cord injury compared to able‐bodied controls (Fig1A and 1B). The level of 4HNE protein adducts was increased at 12 (*P* < 0.05), but not at 1 and 3 months after spinal cord injury (Fig1C and 1D). The amount of GSH (Fig. 1E), as well as of total glutathione (data not shown) was increased at 1 and 3 (*P* < 0.05), but not at 12 months following spinal cord injury. There were no significant differences in the amount of GSSG (Fig. 1F) or the GSH/GSSG ratio (Fig. 1G) between skeletal muscle of spinal cord‐injured individuals and able‐bodied controls. Thus, our results indicate increased lipid peroxidation at 12 months, as well as augmented GSH and total glutathione content in skeletal muscle at 1 and 3 months following spinal cord injury.

### Protein abundance of mitochondrial complexes in skeletal muscle following spinal cord injury

**Figure 2:**
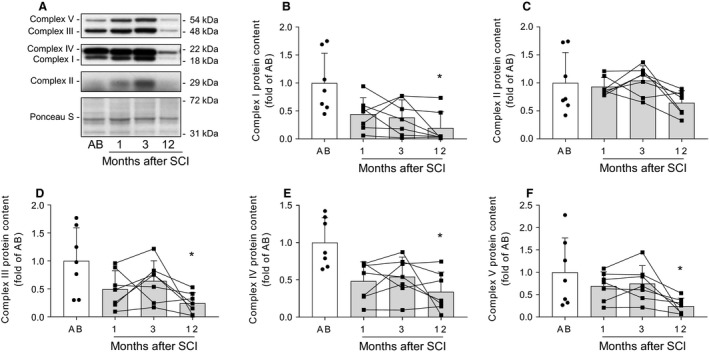
Protein abundance of mitochondrial complexes in skeletal muscle following spinal cord injury. Representative western blot images (A) and quantifications for mitochondrial complex I (B), complex II (C), complex III (D), complex IV (E), and complex V (F) in skeletal muscle from able‐bodied (AB, white bars) and spinal cord‐injured individuals (SCI, grey bars) at 1, 3, and 12 months post‐injury. Units are scaled to the mean of the able‐bodied controls. Data are presented as mean and standard deviation, and individual data points are overlaid. * ‐ *P* < 0.05 vs able‐bodied controls, Kruskal–Wallis followed by Dunn’s multiple comparisons test.

Skeletal muscle mitochondrial content during the first year following spinal cord injury was determined by western blot analysis (Fig. 2A). Protein abundance of mitochondrial complexes I (Fig. 2B), III (Fig. 2D), IV (Fig. 2E), and V (Fig. 2F) was reduced at 12 months (*P* < 0.05), but not at 1 or 3 months after spinal cord injury compared to able‐bodied controls. Conversely, protein levels of mitochondrial complex II were comparable between spinal cord‐injured individuals and able‐bodied controls (Fig. 2C). Overall, abundance of mitochondrial complexes decreased 1 year after spinal cord injury.

### Protein abundance of non‐mitochondrial enzymatic sources of ROS in skeletal muscle following spinal cord injury

**Figure 3:**
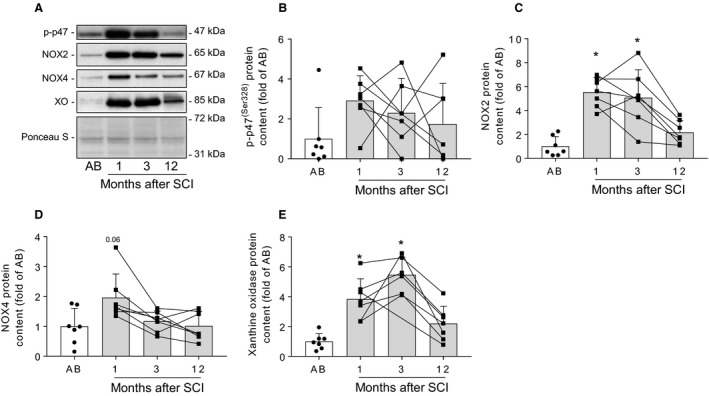
Protein abundance of non‐mitochondrial enzymatic sources of ROS in skeletal muscle following spinal cord injury. Representative western blot images (A) and quantifications for p‐p47 (Ser^328^) (B), NOX2 (C), NOX4 (D), and XO (E) measured in skeletal muscle from able‐bodied (AB, white bars) and spinal cord‐injured individuals (SCI, grey bars) at 1, 3, and 12 months post‐injury. Units are scaled to the mean of the able‐bodied controls. Data are presented as mean and standard deviation, and individual data points are overlaid. * ‐ *P* < 0.05 vs able‐bodied controls, Kruskal–Wallis followed by Dunn’s multiple comparisons test.

To determine whether spinal cord injury leads to changes in non‐mitochondrial ROS production, protein levels of several key enzymes involved in this pathway were analyzed (Fig. 3A). Phosphorylation (Ser^328^) of the p47^phox^‐regulatory subunit of NOX2 was not significantly different between spinal cord‐injured and able‐bodied individuals (Fig. 3B). Conversely, NOX2 protein content was increased at 1 and 3 (*P* < 0.05), but not at 12 months after spinal cord injury (Fig. 3C). Similarly, protein levels of NOX4 tended (*P* = 0.06) to be increased at 1, but not at 3 and 12 months after spinal cord injury (Fig. 3D). Furthermore, protein levels of XO were higher at 1 and 3 (*P* < 0.05), but not 12 months after spinal cord injury (Fig. 3E). Thus, 1 and 3 months after the trauma, spinal cord injury leads to increased protein levels of several non‐mitochondrial ROS‐producing enzymes in the affected skeletal muscle.

### Protein abundance of antioxidant enzymes in skeletal muscle following spinal cord injury

**Figure 4:**
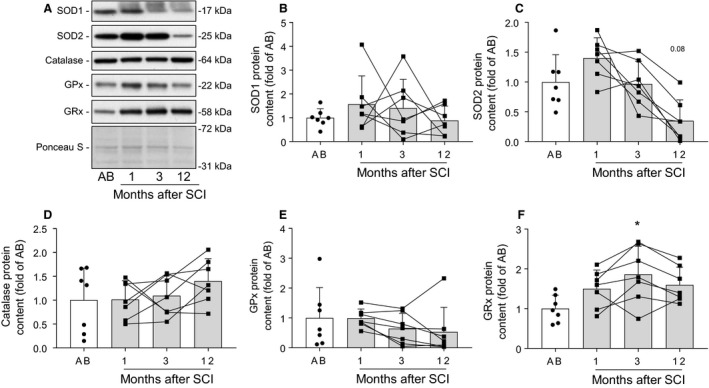
Protein abundance of antioxidant enzymes in skeletal muscle following spinal cord injury. Representative western blot images (A) and quantifications for SOD1 (B), SOD2 (C), catalase (D), GPx (E), and GRx (F) measured in skeletal muscle from able‐bodied (AB, white bars) and spinal cord‐injured individuals (SCI, grey bars) at 1, 3, and 12 months post‐injury. Units are scaled to the mean of the able‐bodied controls. Data are presented as mean and standard deviation, and individual data points are overlaid. * ‐*P* < 0.05 vs able‐bodied controls, Kruskal–Wallis followed by Dunn’s multiple comparisons test.

In order to determine the enzymatic antioxidant defence capacity in skeletal muscle after spinal cord injury, we measured protein levels of several enzymes responsible for ROS detoxification (Fig. 4A). Protein abundance of SOD1 was similar between spinal cord‐injured and able‐bodied individuals (Fig. 4B). Conversely, protein content of SOD2 tended (*P* = 0.08) to be decreased at 12, but not at 1 and 3 months after spinal cord injury (Fig. 4C). Skeletal muscle catalase and GPx protein content were unaltered in spinal cord‐injured versus able‐bodied individuals (Fig. 4D and 4). GRx was increased at 3 (*P* < 0.05), but not at 1 or 12 months after spinal cord injury (Fig. 4F). Overall, our results indicate that protein abundance of antioxidant enzymes is unaltered in skeletal muscle during the first year after spinal cord injury.

### Apoptotic signaling in skeletal muscle following spinal cord injury

**Figure 5:**
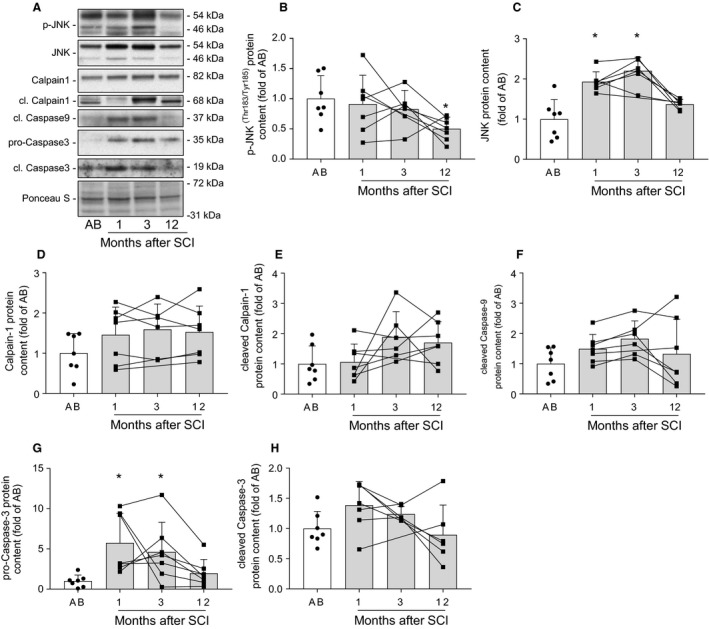
Apoptotic signaling in skeletal muscle following spinal cord injury. Representative western blot images (A) and quantifications for p‐JNK (Thr^183^/Tyr^185^) (B), total JNK (C), calpain‐1 (D), cleaved calpain‐1 (E), cleaved caspase‐9 (F), pro‐caspase‐3 (G), and cleaved caspase‐3 (H) measured in skeletal muscle from able‐bodied (AB, white bars) and spinal cord‐injured individuals (SCI, grey bars) at 1, 3, and 12 months post‐injury. Units are scaled to the mean of the able‐bodied controls. Data are presented as mean and standard deviation, and individual data points are overlaid. *‐*P* < 0.05 vs able‐bodied controls, Kruskal–Wallis followed by Dunn’s multiple comparisons test.

As oxidative stress can lead to an activation of apoptosis, we determined the content of several proteins involved in apoptotic signaling (Fig. 5A). Protein levels of dually phosphorylated (Thr^183^/Tyr^185^) c‐Jun N‐terminal kinase (JNK) were decreased at 12 months (*P* < 0.05), but unaltered at 1 or 3 months after spinal cord injury in comparison to able‐bodied controls (Fig. 5B). Conversely, total protein content of JNK was higher (*P* < 0.05) at 1 and 3, but not at 12 months after spinal cord injury (Fig. 5C). Protein abundance of cleaved caspase‐9, cleaved calpain‐1, and total calpain‐1 did not differ between spinal cord‐injured and able‐bodied individuals (Fig. 5D–F). We detected higher pro‐caspase‐3 protein content at 1 and 3 months (*P* < 0.05), while cleaved caspase‐3 remained unchanged after spinal cord injury (Fig. 5G and H). Overall, our data suggest increased apoptotic signaling in skeletal muscle at 1 and 3 months after spinal cord injury.

## Discussion

**Figure 6:**
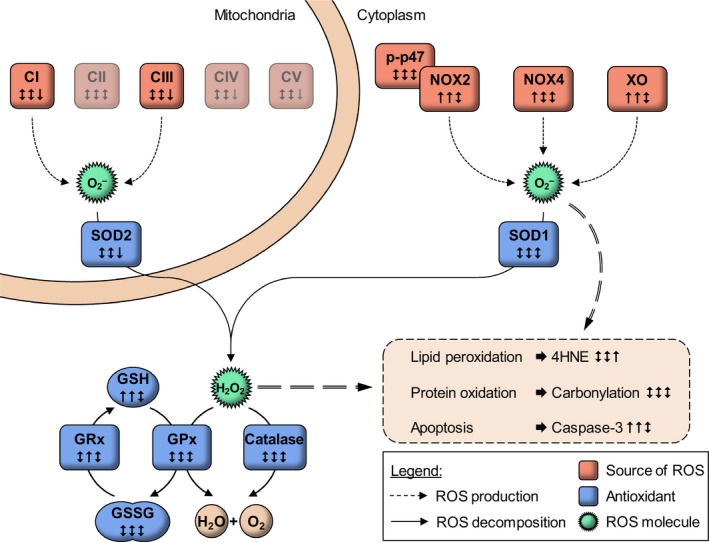
Schematic overview of the investigated pathways and study findings. During the early phase post‐injury, protein abundance of mitochondrial complexes was stable, while enzymatic ROS sources outside the mitochondria and enzymes involved in apoptosis were increased. Concurrently, glutathione content was higher. One‐year post‐injury, most parameters returned to baseline, while mitochondrial protein abundance decreased. Statistical significance is presented in order 1, 3, and 12 months post‐injury compared to able‐bodied controls. ↑ ‐ increased; ↓ ‐ decreased; ↑↓ ‐ not significantly different. Abbreviations used: CI–V (Complex I–V); p‐p47 (Ser^238^ phosphorylated p47^phox^); NOX2 and 4 (NADPH oxidase 2 and 4); XO (xanthine oxidase); SOD1 and 2 ([Cu‐Zn]‐ and [Mn]‐superoxide dismutase); GSH (glutathione – reduced); GSSG (glutathione disulfide – oxidized); GPx (glutathione peroxidase); GRx (glutathione reductase); 4HNE (4‐hydroxynonenal).

We determined whether complete spinal cord injury disturbs ROS homeostasis and induces oxidative stress in human skeletal muscle. A schematic of the main findings and investigated pathways is presented in Figure 6. We detected increased protein abundance of NADPH oxidases and xanthine oxidase at 1 and 3 months after injury, concurrent with increased total and reduced glutathione content and GRx protein levels. Furthermore, our data show increased caspase‐3 content at 1 and 3 months after spinal cord injury suggesting activation of apoptosis. At 12 months following spinal cord injury, we observed increased 4HNE protein adducts and decreased content of mitochondrial complexes and SOD2 protein abundance. Collectively, our study reveals increases in apoptosis and non‐mitochondrial ROS production at 1 and 3 months after spinal cord injury, concomitant with an increase in antioxidant defences.

Mitochondrial ROS production increases in human skeletal muscle during disuse, and the initial phases following spinal cord injury can be accompanied by a transient increase in mitochondrial enzymatic activity (Castro et al., 1999; Gram et al., 2015). Our results indicate unchanged protein abundance of mitochondrial complexes at 1 and 3 months following spinal cord injury. Thus, without changes in mitochondrial protein content, this early phase is likely accompanied by increased mitochondrial ROS production. Conversely, we detected decreased mitochondrial protein content (complexes I, III, IV, and V) at 12 months after spinal cord injury as compared with able‐bodied controls, consistent with our earlier findings (Kostovski et al., 2013). Given that mitochondrial enzymatic activity is decreased (Martin et al., 1992), mitochondrial ROS production is likely lower and thus, probably does not play a major role in the late post‐injury phase.

Several enzymes outside the mitochondria have been implicated in the excessive ROS production in skeletal muscle undergoing atrophy (Kondo et al., 1993; Derbre et al., 2012; Kadoguchi et al., 2018). We detected an increase in XO and NOX2 protein content at 1 and 3 months, and a trend for higher NOX4 protein content at 1 month post‐injury. Increased NOX2 content may reflect higher ROS‐producing capacity during muscle spasms in the early post‐injury phase, as NOX2 is activated by p47^phox^ in response to contraction (Sakellariou et al., 2013). NOX4 protein content increases in rodent skeletal muscle following spinal cord injury and promotes a Ca^2+^‐leak from the endoplasmic reticulum (Liu et al., 2017). Additionally, ROS production by XO is increased in atrophying muscle following hindlimb immobilization, owing to a proteolytic modification of XO by a Ca^2+^‐dependent protease (Kondo et al., 1993). Hence, NOX and XO might form a feed‐forward system increasing ROS production in the early phase of spinal cord injury‐induced muscle atrophy.

Our results suggest that skeletal muscle protein abundance of antioxidant enzymes is retained following spinal cord injury. Unaltered SOD1 and SOD2 protein contents at 1 and 3 months after spinal cord injury indicate that the capacity to decompose superoxide radicals is conserved. Conversely, the decreased abundance of mitochondrial SOD2 at 12 months after injury reflects lower antioxidant protection and mirrors the reduction in mitochondrial content. We found that protein content of GPx and catalase was unaltered between spinal cord‐injured and able‐bodied individuals. Conversely, GRx protein content was elevated at 3 months, and GSH and total glutathione contents were also increased at 1 and 3 months after spinal cord injury. Glutathione biosynthesis is promoted through Akt/mTOR signaling (Kim et al., 2004), which is transiently increased early after spinal cord injury (Lundell et al., 2018). Thus, a compensatory increase in glutathione biosynthesis could account for increased total glutathione content. Together, these results provide evidence suggesting compensatory increases of glutathione antioxidant capacity in skeletal muscle at 1 and 3 months following spinal cord injury.

Protein carbonylation in skeletal muscle of spinal cord‐injured individuals was unchanged compared to able‐bodied controls, while 4HNE protein adducts were increased at 12 months post‐injury. Potentially, the compensatory increases in antioxidants are sufficient to protect proteins from oxidative damage following spinal cord injury. However, proteins damaged by oxidation are preferentially degraded by the ubiquitin‐proteasome system (Shringarpure et al., 2003). We have reported that skeletal muscle atrophy following spinal cord injury is accompanied by increased proteolysis and transient increase in protein synthesis 1 month after spinal cord injury (Lundell et al., 2018). A high protein turnover state, with preferential degradation of oxidized proteins, may lead to underestimation of protein carbonylation. Thus, skeletal muscle may in fact be in a state of oxidative stress in the early post‐injury phase, without detectable increases in carbonylation or 4HNE protein adducts. Conversely, at 12 months post‐injury, skeletal muscle enters a new “steady‐state” of low protein turnover (Leger et al., 2009; Lundell et al., 2018), thereby allowing for accumulation of 4HNE protein adducts.

Excessive ROS exposure and oxidative stress induce apoptosis (Siu et al., 2009). The increase in total JNK protein at 1 and 3 months post‐injury suggests transcriptional and translational mechanisms promoting apoptosis (Fleming et al., 2000). Conversely, the reduction in JNK (Thr^183^/Tyr^185^) phosphorylation and unchanged total protein content at 12 months post‐injury may suggest decreased apoptotic signaling. We did not observe increased cleaved caspase‐9 protein content, indicative of stable mitochondrial apoptotic pathways. However, as caspase‐9 is regulated through the interaction with cytoplasmic factors (Stennicke et al., 1999), the mitochondrial apoptotic pathway may be activated despite the unchanged caspase‐9 protein content. Interestingly, we detected that pro‐caspase‐3 protein content was increased at 1 and 3 months after injury, whereas cleaved caspase‐3 was unchanged following spinal cord injury. Caspase‐3 is an effector caspase (Srinivasula et al., 1998), and an essential protease for inducing protein catabolism in skeletal muscle undergoing atrophy (Du et al., 2004). As caspase‐3 activation by cleavage is a rapid process, we might not be able to observe its significant increase. However, increased JNK and pro‐caspase‐3 protein content suggests increased apoptosis and proteolysis in early stages after spinal cord injury. Although we were unable to discern the specific pathways involved, activation of apoptosis in hindlimb‐unloaded rat skeletal muscle occurs through both extrinsic and intrinsic pathways, despite conserved antioxidant capacity (Andrianjafiniony et al., 2010). Thus, both caspase‐8 and caspase‐9‐dependent pathways may promote apoptosis at 1 and 3 months post‐injury.

The 1‐ and 3‐month time points were chosen based on the rapid skeletal muscle atrophy occurring in the initial 6 weeks post‐injury (Castro et al., 1999). Conceivably, more profound changes could precede the first 1‐month biopsy. However, it was not feasible to obtain biopsies at earlier time points due to the severe nature of the acute phase of spinal cord injury. We have not controlled the overall physical activity of able‐bodied participants, which may account for some variation in the measured parameters. Additionally, we cannot exclude that putative differences in the neurological injury levels or gender exhibit specific oxidative stress responses and might affect some of the parameters measured in this study. Furthermore, due to a relatively low number of participants, we might not be able to detect more subtle alterations. Nevertheless, our study provides valuable insight into the more profound changes in ROS homeostasis in skeletal muscle during the first year following spinal cord injury.

We provide indirect evidence of increased ROS production and apoptotic signaling in the initial rapid phase of spinal cord injury‐induced muscle atrophy. Concurrent increases in glutathione may reflect adaptations to retain redox homeostasis. ROS production is upregulated during skeletal muscle contraction (Ryan et al., 2011; Sakellariou et al., 2013). Therefore, muscular spasms or electrically stimulated rehabilitative exercise may trigger oxidative stress aggravating skeletal muscle atrophy after spinal cord injury. Conversely, contraction‐induced nitric oxide production may compete with superoxide dismutation to hydrogen peroxide by SOD, and promote blood flow in the atrophying muscle (Balon and Nadler, 1994). Thus, investigations into the ROS‐response to such rehabilitative efforts, especially in the initial rapid stage of skeletal muscle atrophy after spinal cord injury, are warranted. Furthermore, treatments with broad ROS scavengers, such as Vitamin E and β‐carotene, or more specific inhibitors of ROS production, such as XO inhibitor allopurinol, have been efficient in alleviating muscle atrophy in rodents and humans (Kondo et al., 1991; Derbre et al., 2012; Ogawa et al., 2013; Ferrando et al., 2018). The development of similar antioxidant adjuvant therapies in the early phase could improve the beneficial responses to rehabilitative interventions and mitigate skeletal muscle atrophy following spinal cord injury.

## Conflict of Interest

No conflict of interest, financial, or otherwise is declared by the authors.
